# “Magic Bullets” at the center stage of immune therapy: a special issue on therapeutic antibodies

**DOI:** 10.1007/s13238-017-0488-1

**Published:** 2017-11-14

**Authors:** Zhiqiang An

**Affiliations:** The Texas Therapeutics Institute, Brown Foundation Institute of Molecular Medicine, University of Texas Health Science Center at Houston, Houston, TX 77030 USA

Therapeutic antibodies represent one of the most significant advances in the history of medicine. Progress in the antibody therapy field was initially slow and intermittent. The first therapeutic antibody, murine-derived Murononab OKT3 for acute organ rejection, was approved by the FDA in 1986, more than a decade after the discovery of the hybridoma technology by Milstein and Köhler in 1975 (Kohler and Milstein, 1975; Emmons and Hunsicker, 1987). As a result of technological breakthroughs starting in the 1980s, progress in therapeutic antibodies accelerated dramatically. During the last 30 years, antibodies have become a major drug modality for cancer, autoimmune, infectious, cardiovascular, neurological, and other diseases. The rapid rise of antibody-based therapies is largely due to their desirable safety profile, target specificity, and efficacy. As a practitioner in the field and like many others, I have stopped from time to time to take the pulse of the therapeutic antibody landscape (An, 2009, 2010). This special issue provides readers with a snapshot of the current state of therapeutic antibodies.

We begin with a comprehensive and expert review on current progress in innovative engineered antibodies by Strohl (2017). Some astonishing statistics were referenced in the review. For instance, more than 74 antibody-based molecules are currently in clinical use and at least 645 antibody based therapies are in different stages of clinical trials. Antibody therapies come in different forms and sizes, such as naked IgGs, antibody drug conjugates (ADCs), bispecific antibodies, Fc fusion proteins, radioimmunoglobulins, and antibody fragments. In addition to the classical targeted therapies such as the HER2 targeting trastuzumab for breast cancer treatment, clinical application of antibody therapies is constantly expanding. Now there are antibodies targeting T cell checkpoints, T-cell redirected bispecific antibodies, and chimeric antigen receptor (CAR) cell-based candidates. Researchers are making progress in new clinical indications and novel disease targets. Further, significant progress is continuously being made on many technology fronts, including new routes of delivery: proteins across the blood-brain barrier; oral delivery to the gut; delivery to the cellular cytosol; and gene- and viral-based delivery of antibodies.

One advantage of antibody based therapies is the long half-life of the molecule. Many factors can influence the pharmacokinetics (PK) of a mAb or Fc-fusion molecule, with the primary determinant being FcRn-mediated recycling. In an expert review on antibody PK, Liu discusses the latest development in enhancing half-life through antibody engineering. He describes the impact of glycosylation, target mediated drug disposition (TMDD), anti-drug antibody (ADA), route of administration, and formulation on antibody PK (Liu, 2017).

Glycosylation plays an important role in the biological activities of antibodies. Manipulation of the glycosylation pattern of an antibody has been used to improve the pharmaceutical properties of the molecule. Mimura et al. summarized the status of applying glycoengineering to improve the safety, functionality, and efficacy of therapeutic antibodies in the era of precision medicine (Mimura et al., 2017).

The antibody-drug conjugate (ADC), an antibody conjugated with potent cytotoxic small molecules through chemical linkers, is an emerging therapeutic format that has great potential to make a paradigm shift in cancer chemotherapy. Tsuchikama and An present an update on the current status in conjugation and linker chemistry design and strategies to develop clinically effective ADCs from medicinal chemistry and pharmacology standpoints (Tsuchikama and An, 2016).

In the oncology area, some of the most exciting new approaches involve antibody modulation of T-cells. Tan et al. reports on the structural basis of durvalumab binding to PD-L1 and the molecular mechanisms of PD-1/PD-L1 blockade. Their study highlights the importance of structural biology in rational drug design (Tan et al., 2017). Bardwell et al. provide an example of using a half DVD-Ig protein format to redirect cytotoxic T lymphocytes (CTLs) to kill tumor cells (Bardwell et al., 2017).

The Fc region of an antibody can recruit effector cells such as natural killer cells, macrophages, or neutrophils. It can also activate the complement system to destroy the target-associated cells. These properties referred to as “antibody-dependent cell cytotoxicity (ADCC)” and “complement-dependent cytotoxicity (CDC)”, respectively, are fundamental aspects of antibody biology that are being manipulated to create therapeutics with more potent biological activities. Wang et al. provided an overview on various antibody engineering efforts intended to improve efficacy and safety relative to the human IgG isotype (Wang et al., 2017b).

Antibodies are an important component in host immune responses against viral infections. It is natural that antibodies are becoming a viable therapeutic modality for treatment of viral infections including emerging viral pathogens such as Ebola that represent heightened public health concerns, as well as pathogens that have long been known, such as HBV. Kang et al. describe the preclinical PK study of an anti-HBV humanized and Fc-modified monoclonal antibody in mice and nonhuman primates (Kang et al., 2017).

Unlike small-molecule based therapies, therapeutic antibodies are large, complex molecules that are not easily formulated or delivered. In addition, therapeutic antibodies are produced as heterogeneous mixtures of molecules including different glycoforms that can vary greatly in molecular structure. Wang et al. reviewed the complex analytical tools that have been developed and optimized for the molecular and functional characterization of antibody therapeutics (Wang et al., 2017a). The basic principles of evaluation of biosimilar antibody therapies are discussed in the light of recommendations by the World Health Organization (WHO).

Despite the many advantages of antibodies as a drug modality, they have several limitations, chief amongst those being the high cost of manufacture. Therefore, non-antibody binding proteins have long been sought after as alternative therapies. Simeon and Chen provide an update on protein scaffolds that are being investigated and developed as therapeutic alternatives to antibodies (Simeon and Chen, 2017). Advantages and limitations of these protein scaffolds as therapeutics compared to antibodies are the subjects of a comprehensive discussion.
